# High-Pressure Processing for Anisakis Larvae Inactivation: Fish Quality Changes and Near-Infrared Spectroscopy to Verify Its Application

**DOI:** 10.3390/foods15071218

**Published:** 2026-04-03

**Authors:** Marzia Pezzolato, Alberto Brugiapaglia, Riccardo Provera, Francesco Gai, Jacopo Pio Salvatico, Francesco Pennisi, Nunzia Giaccio, Alfredo Greco, Elena Bozzetta, Giovanna Esposito

**Affiliations:** 1 Istituto Zooprofilattico Sperimentale del Piemonte, Liguria e Valle d’Aosta, Via Bologna 148, 10154 Torino, Italy; marzia.pezzolato@izsplv.it (M.P.); francesco.pennisi@izsplv.it (F.P.); nunzia.giaccio@izsplv.it (N.G.); elena.bozzetta@izsplv.it (E.B.); giovanna.esposito@izsplv.it (G.E.); 2 Department of Agricultural, Forest and Food Sciences, University of Turin, 10095 Grugliasco, Italy; alberto.brugiapaglia@unito.it (A.B.); jacopopio.salvatico@unito.it (J.P.S.); 3 CNR Institute of Science of Food Production, Turin Division, 10095 Grugliasco, Italy; francesco.gai@cnr.it; 4 Dipartimento di Sanità Pubblica, Azienda Unità Sanitaria Locale di Parma, Via Vasari, 43126 Parma, Italy; algreco@ausl.pr.it

**Keywords:** food safety, Anisakis, fish quality, near-infrared spectroscopy, chemometrics, LDA, SVM

## Abstract

The increasing consumption of raw and minimally processed fish products has raised concerns regarding the risk of anisakiasis, the infection caused by ingesting larvae of the *Anisakis* genus. Freezing is currently the standard control measure; however, alternative non-thermal technologies are being explored to preserve product quality while ensuring safety. Several studies have investigated the impacts of high-pressure processing (HPP) on seafood products, but limited information is available about the minimum effective pressure required to achieve complete inactivation of *Anisakis* larvae while maintaining fillet quality. Moreover, no studies have evaluated the use of portable near-infrared (NIR) spectroscopy as a rapid tool to authenticate HPP-treated fish products. This study evaluated the efficacy of HPP in inactivating *Anisakis* spp. larvae in gilthead sea bream (*Sparus aurata*) fillets and investigated the impact of treatment on physicochemical quality parameters. In addition, the reliability of portable NIR spectroscopy coupled with chemometrics was assessed for rapid discrimination between treated and untreated samples. HPP treatments were applied with different pressure–time combinations, and the treatment at 200 MPa for 5 min was selected as the optimal treatment since it was able to achieve 100% larval inactivation. Quality evaluation showed significant changes in color (increase in L* values) and texture parameters, consistent with pressure-induced denaturation, while lipid oxidation remained within acceptable limits. NIR spectra analysis combined with chemometrics approach allowed discrimination between not treated and HPP-treated fillets with an overall accuracy of 98%. The results demonstrate that HPP at moderate pressure levels represents a promising alternative to freezing for *Anisakis* larvae inactivation in farmed sea bream, and that portable NIR spectroscopy may serve as a rapid, non-destructive tool for on-site verification of treatment. This combined approach could support the development of innovative control strategies in seafood safety management

## 1. Introduction

The growing consumption of raw fish products has raised significant concerns regarding food safety. Although the intake of raw foods may offer nutritional advantages, such as reduced protein denaturation, improved vitamin retention, and preservation of organoleptic properties, it also increases the risk of foodborne diseases caused by pathogenic bacteria and parasites [1].

*Anisakis* spp. is a fish-borne parasitic nematode whose larvae (2–5 cm in length) are visible to the naked eye [2] and detectable through careful visual inspection [3]. Their complex life cycle involves multiple hosts, including crustaceans, cephalopods, fish, and marine mammals [4]. Third-stage larvae (L3), which normally parasitise adult marine mammals, can accidentally infect humans following the consumption of raw or inadequately cooked fishery products, causing anisakiasis. This zoonotic disease may occur with gastrointestinal, allergic, or granulomatous manifestations, including abdominal pain, vomiting, and hematemesis, often leading to diagnostic challenges [1,4,5,6]. Over the last five decades, the global incidence of anisakiasis has increased considerably, emerging as a relevant public health concern [7]. As of 2024, approximately 5000 human cases have been reported worldwide, particularly in endemic areas such as Japan, China, Thailand, and other Southeast Asian countries, as well as Mexico, Colombia, and Peru [8]. Japan reports the highest number of cases, with 2000–3000 infections annually, mainly associated with the consumption of sushi and sashimi [3]. In Europe, Spain shows the highest incidence, followed by Italy [4]. Nevertheless, the true burden of anisakiasis is likely underestimated, especially in Mediterranean countries where parasitized fish are highly prevalent [9].

In Italian seawaters, the fish species most frequently infected by *Anisakis* spp. include sardine, whiting, horse mackerel, anchovy, mackerel, cod, monkfish, Peter’s fish, and silver scabbardfish [10].

To mitigate the risk of infection, different countries have established regulations or guidelines for parasite control in fishery products. In the United States, effective larval devitalization requires freezing at −35 °C for 15 h or −20 °C for 7 days [10]. Regulation (EC) No 853/2004 lays down specific hygiene rules for food of animal origin and provides that, to ensure food safety, fishery products intended to be eaten raw must either be frozen at −20 °C for at least 24 h (or at −35 °C for at least 15 h) or be subjected to alternative treatments of proven efficacy (e.g., adequate heat treatment or marinating) [5,11].

Conventional freezing treatments are primarily focused on ensuring complete larval inactivation throughout the entire product mass; however, their impact on product quality cannot be entirely excluded [12]. Therefore, the evaluation of alternative inactivation strategies is of increasing interest. Among emerging technologies, high-pressure processing (HPP has gained attention as a non-thermal method capable of ensuring microbiological safety while preserving product quality. HPP consists of subjecting packaged food products to hydrostatic pressures typically ranging from 100 to 600 MPa in a water-filled vessel for defined holding times. Pressures of 400–600 MPa effectively inactivate common seafood pathogens such as *Vibrio* and *Listeria* spp., while pressures up to 1200 MPa have been investigated for spore inactivation [13,14,15]. However, depending on treatment intensity and fish species, HPP may induce textural changes and a cooked-like appearance, potentially limiting its industrial application [16].

The effects of HPP on seafood can be effectively monitored using rapid and non-destructive spectroscopic methods, thus supporting food safety management. These techniques, including NIR spectroscopy, mid-infrared spectroscopy (MIR), hyperspectral imaging (HSI), and nuclear magnetic resonance (NMR) spectroscopy, are increasingly applied in food analysis for rapid and non-destructive quality assessment [17]. Among these, NIR spectroscopy represents a rapid, cost-effective, and non-invasive analytical tool capable of generating chemical fingerprints of complex food matrices. The development of portable and miniaturized NIR devices further enables on-site applications in food processing environments [18]. Coupled with chemometric methods, NIR spectroscopy allows both qualitative and quantitative characterization of food systems through multivariate data analysis [18,19,20].

Principal Component Analysis (PCA) is commonly used for exploratory analysis of spectral datasets, as it helps to reduce dimensionality and reveal underlying patterns. In contrast, supervised classification methods, such as Linear Discriminant Analysis (LDA) and Support Vector Machines (SVM), are typically applied for discrimination tasks, such as distinguishing between different seafood species in authentication studies [21].

Despite its widespread use in seafood preservation, limited information is available regarding the effectiveness of HPP against *Anisakis* spp. larvae. While several studies have evaluated the impact of HPP on color and texture of seafood products, only a few have investigated its larvicidal effect, and no spectroscopic approach has been applied to monitor treatment efficacy in fish matrices [22,23].

Therefore, the present study aimed to (i) evaluate the efficacy of high-pressure processing (HPP) in inactivating *Anisakis* spp. larvae in artificially infested fish, (ii) assess potential quality changes induced by the treatment, and (iii) investigate the use of a portable VIAVI MicroNIR device combined with multivariate analysis as a rapid tool to distinguish treated from untreated samples. Sea bream was selected as the model species due to its commercial importance in Mediterranean aquaculture and its low natural prevalence of *Anisakis* spp., which enabled controlled artificial inoculation and reliable evaluation of larval inactivation [11].

## 2. Materials and Methods

### 2.1. Sample Collection and Artificial Infestation

*Anisakis* spp. larvae were isolated from the visceral cavity of non-eviscerated Atlantic mackerel (*Scomber scombrus*) (300–400 g) purchased fresh from local fish markets. After extraction, larvae were placed in a 1% acetic acid solution to assess viability and motility before artificial infestation.

Fresh fish fillets (n = 200) of farmed sea bream, originating from the Mediterranean area, were acquired from supermarkets. Fillets were previously inspected and confirmed to be free of *Anisakis* spp. larvae before experimental use.

Artificial infestation was performed prior to HPP treatment. Each fillet was inoculated with three live larvae using a porcupine needle to ensure standardized insertion into the muscle tissue. Samples were vacuum-packaged and stored at 4 °C for 12 h before processing.

All fish samples used in the study were not alive at the time of sampling.

### 2.2. Viability Determination

Once isolated from fish fillets, the *Anisakis* spp. larvae were transferred to a Petri dish containing 1% acetic acid solution (*v*/*v*) and visually inspected for viability. Observations were made at room temperature under a stereomicroscope (VWR VisiScope IT404, VWR International S.r.l., Milan, Italy), both immediately and after 1 h and 24 h. Larvae were carefully stimulated with dissecting needles, and the lack of visible movement or spontaneous reaction was interpreted as loss of viability.

### 2.3. Experimental Design

Fish fillets (mean weight: 120 g) were randomly divided into two experimental groups (Table 1):

Group 1: larval inactivation was evaluated under four different high-pressure treatments, and NIR spectra were acquired from all samples.

Group 2: Based on the minimal visual impact observed at 200 MPa in Group 1, additional experiments were conducted at this pressure value to further evaluate NIR spectra, larval inactivation and physicochemical properties.

Therefore, Group 1 was used to evaluate the effects of different HPP treatments and explore spectral differences, while Group 2 focused on the selected treatment (200 MPa for 5 min) for further physicochemical characterization and classification modeling.

### 2.4. HPP Treatment

HPP treatments were performed using industrial-scale equipment (AV-40X, JBT Avure, Erlanger, KY, USA) equipped with a horizontal cylindrical pressure vessel (total volume: 525 L; internal diameter: 471 mm; internal length: 3000 mm). The temperature increase induced by pressurization (adiabatic heating of water-based systems) was approximately 3 °C per 100 MPa, corresponding to an increase of ~6 °C at 200 MPa, 9 °C at 300 MPa, and 18 °C at 600 MPa. Considering an initial product temperature of 4 °C, the maximum temperature reached at 600 MPa was approximately 22 °C. Pre-compression temperature was maintained at 4 ± 1 °C using a water circulation system (cooling water flow rate: 300 L min^−1^ at 1.0 °C). Vacuum-packaged samples, along with distilled water, were loaded into the pressure container, which was pressurized for 1, 3 or 5 min (holding time) to pressures of 200, 300 or 600 MPa. The pressure build-up velocity was approximately 150 MPa per min, typical of large-volume industrial HPP systems, and the decompression time was less than 10 s.

After treatment, fish samples containing *Anisakis* spp. larvae were stored at 4 °C until further analysis. Larvae were subsequently recovered from each sample and transferred to Petri dishes containing 1% aqueous acetic acid solution. Spontaneous motility was monitored for 24 h, after which larval viability was determined.

### 2.5. Physicochemical Analysis

#### 2.5.1. Purge Losses

For the determination of purge losses (PL), fish fillets were first weighed in the bag (W_1_) and after removal from the bag (W_3_). Finally, the bag was rinsed, dried, and weighed (W_2_) and PL was calculated as shown in Equation (1):

(1) $$PL\left(\%\right)=\frac{W_{1}-\left(W_{2}+W_{3}\right)}{W_{1}-W_{2}}\cdot 100$$

#### 2.5.2. pH

The ultimate pH was determined using a portable pH-meter (PH25+, Crison Instruments S.A., Alella, Spain) equipped with a penetration probe. The device was calibrated for muscle temperature prior to each measurement using pH 4.0 and 7.0 buffers. Measurements for pH were conducted at 3 separate locations, and the average of these measurements was reported.

#### 2.5.3. Color

Instrumental color measurements were determined on the dorsal surface of each fillet at three different locations using a spectrophotometer (CM-600d, Konica Minolta sensing Inc., Osaka, Japan) equipped with an 8 mm diameter aperture. The device was set with the Specular Component Excluded, D_65_ light source, 8° viewing angle and 10° standard observer. The instrument was standardized with a provided white standard plate prior to use. The results were expressed according to the Commission International de l’Eclairage (CIE) system [24] and reported as L* (lightness), a* (redness), and b* (yellowness), Chroma (C*) and Hue angle (h°). The Chroma was calculated as shown in Equation (2), and the Hue angle as shown in Equation (3).

(2) $$C^{*}=\sqrt{{\left(a^{*}\right)}^{2}+{\left(b^{*}\right)}^{2}}$$

(3) $$h^{{}^{\circ}}=\tan^{-1}\left(\frac{b^{*}}{a^{*}}\right)$$

Chroma represents the saturation or vividness of a color. Hue angle refers to degree of the dominant spectral components such as red, green, and blue and ranges from 0° to 360°. In addition, the color Whiteness Index (WI) was estimated by Equation (4) [25]:

(4) $$WI=100-\sqrt{{{\left(100-L^{*}\right)}^{2}+\left(a^{*}\right)}^{2}+{\left(b^{*}\right)}^{2}}$$

#### 2.5.4. Textural Properties

The textural properties of the fish fillet were evaluated using a texture analyzer (TA.HD plusC, Stable Micro Systems, Surrey, UK) in combination with a 10 mm diameter spherical probe at three different locations in the dorsal side of each fillet. During the TPA test, the sample undergoes a 2-cycle uniaxial compression to 50% of its original height with a 1 sec recovery period between compressions. The movement speed of the probe was 200 mm/min. Analysis of the force-time curve lead to the following textural parameters: Hardness (N), Springiness (ratio), Adhesiveness (Nxs), Cohesiveness (ratio), Gumminess (N) and Chewiness (N) [26]

#### 2.5.5. Water Holding Capacity

Water holding capacity (WHC), the ability of muscle to retain water, was determined using two methods: (1) a low-speed centrifugation method [27] modified by adjusting the applied centrifugal force; and (2) by filter paper press method [28,29]. To determine centrifugation loss, raw fish sample was cut into pieces weighing approximately 2 g (±0.1 g) and placed on a fine screen, to prevent reabsorbing of expelled water, in a 50 mL centrifugation tube. Samples were centrifuged for 10 min at 2500 rpm. WHC of the raw samples was then calculated as the percentage of water remaining relative to the initial water content, using the following Equation (5):

(5) $$WHC\left(\%\right)=\frac{W_{0}-\Delta r}{W_{0}}\cdot 100$$

where

W_0_ = water content of the sample before centrifugation (%);

$\Delta_{r}$ is the weight lost by centrifugation (%) and was calculated by Equation (6):

(6) $$\Delta_{r}=\frac{W_{1}-W_{2}}{W_{1}}\cdot 100$$

W_1_ = weight of the original sample (g)

W_2_ = weight of the sample after centrifugation (g)

The water-holding capacity (WHC) of the sample was also calculated according to the following Equation (7) [29]:

(7) $$WHC\left(\%\right)=\frac{\left(m\cdot w)-(100\cdot d\right)}{\left(m\cdot w\right)}\cdot 100$$

where:

m is the mass of the sample before WHC determination (mg), 

w is the water content of the sample before WHC determination (%), 

d is the difference in the mass (mg) of the sample before and after loading.

#### 2.5.6. Water Activity

The water activity (aw) measurements were carried out by using a water activity meter (AcquaLab PRE, METER Group Inc., Pullman, WA, USA) at 20 °C. Approximately 5 g of a homogeneous fish sample was put in a disposable sample cup, completely covering the bottom of the cup and filling not more than half of the cup. The cup was placed into the drawer and securely closed. After 3 min, sample measurements of water activity were directly calculated by the instrument and recorded.

#### 2.5.7. Electrical Conductivity

The electrical conductivity (EC; in μS/cm), was measured at room temperature using a conductivity meter (HI6522, Hanna Instruments, Woonsocket, RI, USA) following a slightly modified method [30]. Approximately 5 g of ground sample was mixed with 50 mL of deionized water and stirred for 30 min. The mixture was filtered by a medium-speed qualitative filter paper, and the EC of the filtrate was directly measured. A conductivity electrode was then inserted until a stable reading was obtained. The instrument was calibrated before use with standard conductivity solution in the concentration range of 1410 and 5000 μS/cm.

#### 2.5.8. Proximate Composition

Each fillet, after skin removal, was homogenized with a grinder (Moulinette DPA141, Moulinex, Groupe SEB, Écully, France) for subsequent proximate composition. Moisture was determined gravimetrically by the difference between the wet weight and the dry weight of the sample. The sample was oven dried to a constant weight (125 °C, 5 h) according to AOAC method 950.46 [31]. Dry sample obtained after moisture analyses was used for ash determination. Dry meat sample was ashed in a muffle furnace at 550 °C, until a white ash was obtained, using AOAC method 920.153 [31]. The nitrogen content was determined by means of the Kjeldahl method (method 928.08 [31] using a Büchi Distillation Unit K-355 (BÜCHI Labortechnik AG, Flawil, Switzerland), and a nitrogen-to-protein conversion factor of 6.25 was used to calculate the protein content. The fat content was determined by means of Soxhlet extraction, using a Büchi Extraction System B-811 (BÜCHI Labortechnik AG, Flawil, Switzerland) with petroleum ether, according to AOAC method 991.36 [31]. The proximate composition results were expressed as g/100 g fish muscle and presented as average values of two replicates.

#### 2.5.9. Lipid Oxidation

After HPP treatment, 20 fillets (10 T and 10 NT) were removed from their vacuum packaging, placed in trays covered with cling film, and stored under aerobic conditions at 4 °C for up to seven days. The secondary lipid oxidation was determined using the modified thiobarbituric acid (TBA) procedure [32]. Five grams of fish meat was mixed and homogenized for 30 s with 10% (*v*/*v*) trichloroacetic acid (TCA) using a Polytron tissue homogenizer (Type PT 10–35; Kinematica GmbH, Luzern, Switzerland). The supernatant was filtered through Whatman #1 filter paper. One milliliter of filtrate was combined with 1 mL of a 0.02 M aqueous 2-TBA solution, heated in a boiling water bath for 20 min together with a blank containing 1 mL of a TCA/water mix (1/1) and 1 mL of a TBA reagent and subsequently cooled under running tap water. The samples were analysed in duplicate, and the absorbance was read at 532 nm with a Helios spectrophotometer (Unicam Limited, Cambridge, UK) against a blank that contained all the reagents, but no fish sample. TBARS values were calculated from a standard curve of 1,1,3,3-tetramethoxypropane (TMP; Sigma–Aldrich, Steinheim, Germany) and expressed as mg TMP/kg of fish meat.

### 2.6. Statistical Analysis

Data obtained from physicochemical analyses were preliminarily assessed for normality and homogeneity of variances using the Shapiro–Wilk and Levene’s tests, respectively.

When both assumptions were satisfied, differences between NT and T were evaluated by one-way analysis of variance (ANOVA) using the General Linear Model (GLM) procedure in SPSS (IBM SPSS Statistics, version 21.0; IBM Corp., Armonk, NY, USA), considering HPP treatment as fixed factor. Post hoc comparisons were performed using Tukey’s honestly significant difference (HSD) test. Statistical significance was set at *p* < 0.05.

When the normality assumption was not met, comparisons were performed using the Mann–Whitney U test. No data transformation was applied.

### 2.7. NIR Acquisition and Multivariate Analysis

NIR spectra were acquired using a handheld MicroNIR device (VIAVI Solutions Inc., Milpitas, CA, USA) in the wavelength range 908–1676 nm. Instrument control and spectral acquisition were managed through VIAVI MicroNIR Pro software (v3.2) operated on a laptop computer. Spectral data were exported in ASCII format and processed using The Unscrambler^®^ software (version 10.4.1; CAMO Software AS, Oslo, Norway). For each fillet, three replicate spectra were collected at different surface locations and averaged prior to analysis. All spectra were mean-centered and mathematically pre-treated by baseline and standard normal variate (SNV) to correct light scattering effects before chemometrics analysis.

Two chemometric strategies were applied: an unsupervised approach based on PCA, and supervised classification models using LDA and SVM algorithms.

PCA was applied to Group 1 samples to investigate separation according to different HPP treatments. LDA and SVM were applied to build classification models for discriminating between treated (T) and untreated (NT) fish samples. Three parameters were selected to evaluate the models’ performance: specificity, sensitivity, and accuracy [33]. Group 2 samples were randomly divided into a training set (80%) and an independent validation set (20%). The training set was used to build the classification models, whereas the validation set was employed to assess their performance and predictive capability. For the SVM model, hyperparameters were optimized on the training set using the software’s internal cross-validation procedure. The kernel type (radial basis function) and parameters (C = 1 and γ = 0.008) that provided the best classification performance were retained for the final model.

## 3. Results

### 3.1. Inactivation of Anisakis spp. Larvae by HPP Treatment

All tested HHP conditions resulted in complete larval inactivation (100%). In contrast, viable *Anisakis* larvae were consistently recovered from artificially infested control samples that were not subjected to HPP treatment, confirming the effectiveness of the pressure treatments.

### 3.2. Physicochemical Quality Attributes of HPP-Treated Fillets

**Figure 1:**
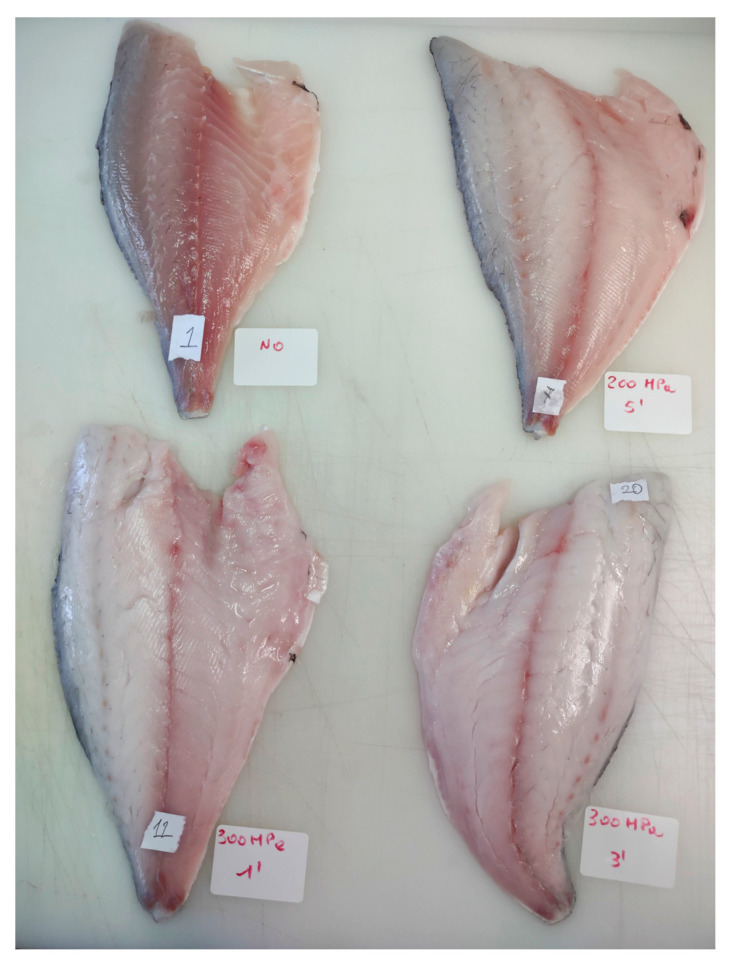
Gilthead sea bream fillets subjected to different high-pressure processing (HPP) treatments. Top left: untreated control; top right: 200 MPa for 5 min; bottom left: 300 MPa for 1 min; bottom right: 300 MPa for 3 min. The photo illustrates the visual effects of HPP on fillet appearance, including changes in color.

Differences in physicochemical parameters between T and NT samples are reported in Table 2. Results refer to Group 2 samples, as the 200 MPa treatment—selected based on Group 1 outcomes achieved complete larval inactivation while showing the lowest visual impact (Figure 1).

The mean pH of NT fillets was 6.24, consistent with values reported for fresh sea bream stored in melting ice [34], thus confirming the initial freshness of the samples. Following HPP at 200 MPa, pH increased significantly to 6.31 (*p* < 0.05), in agreement with previous studies [35,36].

A significant increase (*p* < 0.05) of approximately 50% in purge loss was observed after treatment, along with a significant variation in water-holding capacity (WHC). In contrast, electrical conductivity did not differ significantly between T and NT samples (*p* > 0.05). Water activity (aw) decreased slightly from 0.941 to 0.914; however, this reduction was not statistically significant (*p* > 0.05).

HPP significantly affected colour parameters. Chroma (C*) increased from 2.92 to 7.00 (*p* < 0.05), indicating a more vivid appearance compared with the duller NT samples (Figure 1). T fillets showed a significant reduction in hue angle (h°), associated with a shift toward yellow tones (increase in b* values), and a significant increase in lightness (L*), resulting in a characteristic light yellow–grey, cooked-like appearance (*p* < 0.05). The whiteness index also increased significantly after treatment (*p* < 0.05).

Regarding proximate composition, HPP at 200 MPa induced only minor, non-significant changes in fish muscle components.

Texture profile analysis revealed significantly lower hardness and gumminess values and higher springiness and cohesiveness in T samples compared with NT (*p* < 0.05). Adhesiveness and chewiness were not significantly affected by pressurization.

Lipid oxidation, evaluated through thiobarbituric acid reactive substances (TBARS) as an index of malondialdehyde formation, showed significantly lower values in HPP-treated fillets compared with NT fillets (*p* < 0.05), in agreement with previous findings [37].

### 3.3. NIR Spectral Features and Multivariate Discrimination

**Figure 2:**
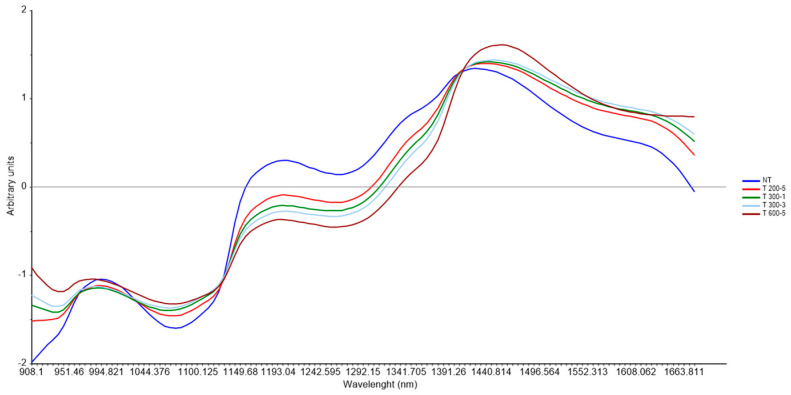
Mean pre-processed NIR spectra of *Sparus aurata*. Each color represents a different experimental condition: untreated samples (NT) and samples subjected to different treatments (T).

Figure 2 shows NIR pre-processed spectra of T and NT samples. Looking at the spectra, the most prominent band corresponds to the first overtone of the O-H group, arising from water and chemical groups bonded with water via hydrogen bonds (ca. 1450 nm). The band at ca. 1000 nm can be attributed to protein -NH2 groups, while the signal between around 1200 nm is related to the absorbance of CH, CH2, and CH3 groups, related to the second overtone of C-H stretching in lipids [21].

**Figure 3:**
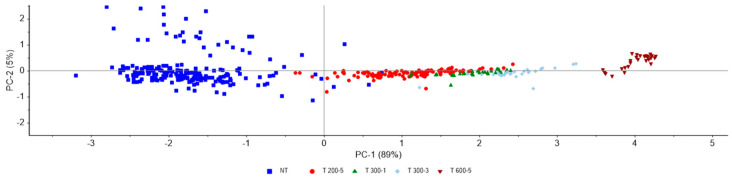
Two-dimensional PCA score plot of sea bream at different treatments: NT (blue), HPP 200 MPa for 5′ (red), HPP 300 MPa for 1′ (green), HPP 300 MPa for 3′ (light blue), HPP 600 MPa for 5′ (maroon).

The PCA score plot of Group 1 (Figure 3) shows clear separation of samples according to HPP treatment, with NT samples forming a distinct group. PC1 and PC2 explain the majority of variance 89% and 5%, respectively.

**Figure 4:**
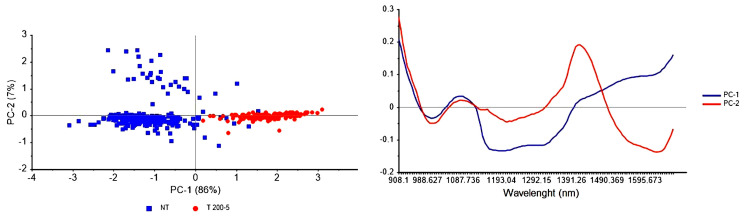
Two-dimensional PCA score plot of NT (blue) and HPP treated at 200 MPa 5′ (red) respectively (on the left) and the corresponding loading plot (on the right). PC1 and PC2 explained respectively 86% and 7% of the total variance. Loading plot of PC1 and PC2 showing the contribution of each wavelength separation observed in the scores plot; high absolute values highlight key regions, particularly around 1450 nm, corresponding to the water absorption band.

Results from Group 1 samples showed that PCA can separate T and NT samples. Even the lowest HPP condition (200 MPa for 5 min) allowed good separation between T and NT samples (Figure 4) without drastically altering the visual appearance of the fish (Figure 1). Preservation of organoleptic and nutritional properties is an important aspect for consumers and represents the main advantage of the high-pressure process for modern food production [38].

Considering the reduced visual impact derived from the 200 MPa treatment, experiments on Group 2 samples were performed at that pressure.

**Figure 5:**
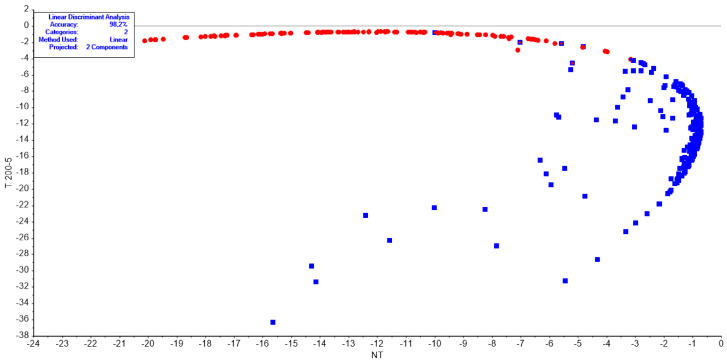
LDA of fish samples not treated and treated with HPP at 200 MPa for 5 min. The plot illustrates the separation of the two sample groups, achieving a classification accuracy of 98%.

The LDA and SVM models built to classify T vs. NT samples showed accuracy higher than 98% (Table 3). As illustrated in Figure 5, the LDA score plot highlights a clear separation between the two groups. The specificity is related to the ability to avoid false positives (i.e., T samples wrongly classified as NT). Sensitivity (Sens) is the ability to avoid false negatives (i.e., NT samples wrongly classified as T).

## 4. Discussion

### 4.1. Effect of HPP Treatment on the Viability of Anisakis spp.

Over the last two decades, the application of high hydrostatic pressure in food processing has increased substantially, thanks to its effectiveness in inactivating pathogens while preserving product quality. Indeed, preservation of organoleptic and nutritional properties is an important aspect for consumers and represents the main advantage of the high-pressure process for modern food production [38]. Results of the present study highlight that all tested pressure levels resulted in complete death of larvae, suggesting that even mild treatment (200 MPa) is sufficient to achieve this objective.

These findings are consistent with previous research reporting complete inactivation of *Anisakis* spp. larvae following HPP treatment at 200 MPa for 10 min at 0 and 15 °C [39]. These results support HPP as a promising alternative strategy for effective parasite inactivation in fishery products.

The combination of moderate pressure, controlled holding time, and temperature used in this study not only ensured complete larval inactivation but also contributed to the preservation of key quality attributes such as visual appearance and texture. Unlike extreme freezing or prolonged thermal treatments, HPP achieves inactivation in significantly shorter processing times and at lower energy input, making it an efficient and practical option for industrial applications in the seafood sector.

### 4.2. Effect of HPP on Physicochemical Characteristics of Sea Bream Fish Fillets

T sea bream fillets exhibited higher pH values compared with NT samples. Several mechanisms have been proposed to explain the increase in pH observed in pressurized samples. Some authors have suggested that the pH increase is associated with protein denaturation, which becomes more intense between 300 and 400 MPa, probabily due to the partial or total denaturation of myofibrillar and sarcoplasmic proteins in fish muscle. Changes in the tertiary and quaternary structures may expose alkaline amino acid residues, such as the imidazole group of histidines, which can ionize and shift the medium toward alkalinity. Similarly, acid radicals may become less available, further contributing to the observed pH increase [40,41].

Numerous studies suggest that HPP typically increases purge loss, as pressure induces denaturation of myofibrillar proteins, disrupts cellular membranes, and compromises the integrity of the muscle matrix responsible for water retention, particularly at moderate to high pressures (e.g., 200–600 MPa). Pressures above ~200–300 MPa promote the unfolding of key structural proteins, such as myosin and actin, reducing their water -binding capacity and leading to increased drip/purge during refrigerated storage [42].

Free water accounts for approximately 90% of the total moisture in fish tissue and is mainly located intracellularly. Its distribution is influenced by protein structural changes, redistribution of fluid between intra- and extracellular compartments, pH, and ionic strength [43,44]. The reduction in water-holding capacity (WHC) observed after HPP treatment may be attributed to fiber compression and protein denaturation [40]. Pressure-induced denaturation of structural proteins, particularly actin and myosin, alters the myofibrillar architecture and reduces the ability of muscle cells to retain water [40,45,46]. Myosin in various fish species denatures between 100 and 200 MPa, actin around 200 MPa, whereas sarcoplasmic proteins are more pressure-resistant and may remain stable up to 400 MPa [47]. WHC is a critical quality parameter in fish, as it directly influences textural and sensory attributes. In the present study, WHC showed a statistically differences between NT and HPP T samples. In sea bass fillets, WHC decreased at higher pressures (250 and 400 MPa) compared with 100 MPa treatment [48], whereas other studies reported a slight increase up to 300 MPa followed by a progressive decrease with increasing pressure [49].

Understanding the effect of HPP on fish colour is essential, as colour is a primary indicator of freshness and a key factor in consumer purchasing decisions [37,40]. Given that HPP significantly alters protein structures, changes in colour and colour stability are expected [50]. Our results are consistent with the findings reported in previous studies [25]. The increase in lightness (L*) could be a function of the treatment intensity, in particular the combination of pressure, duration, and temperature, which causes the denaturation of globin and myofibrillar proteins [51]. Although some variability exists among studies, most report a decrease in redness (a*) and stable or increased yellowness (b*), depending on species and processing conditions [40,52]. The increase in b* values in pressurized fish occurs due to the effects of pressure on proteins, with a mechanism similar to that which influences L* values and whiteness of the samples [51]. Since fish muscle is characterized by low myoglobin content, increased light scattering due to denaturation of myofibrillar proteins (mainly myosin and actin) likely explains the observed increase in L* and whiteness [37,40]. A significant increase in whiteness was also reported in sea bass muscle treated at 100 MPa (10 °C for 5 min) compared to control samples [49] and also L* values and whiteness index (WI) were positively correlated with both pressure level and holding time [53]. More intense treatments resulted in higher L* and WI values, conferring a “cooked-like” appearance to the fillets [54].

Texture is a crucial quality attribute affecting fish product palatability, and fillet softening represents a significant issue for the fish industry. Therefore, texture profile analysis (TPA) is a valuable tool for evaluating the effects of HPP on fish muscle quality [55]. High-pressure processing is generally associated with increased hardness, and the magnitude of these changes is directly related to pressure intensity and aggregation or denaturation of myofibrillar proteins such as actin and myosin [40,42,52].

Depending on pressure level, HPP induces several molecular phenomena, including oligomer dissociation, protein unfolding, denaturation, aggregation, and gelatinization, all of which contribute to the final textural profile [56,57,58]. Pressurization time and the intrinsic chemical composition of the fish also influence texture modifications [59]. While treatments at 500–600 MPa have been shown to increase hardness in gilthead sea bream and sea bass [49,52], lower pressures may have the opposite effect. Sea bass pressurized at 100, 200, and 300 MPa exhibited significantly reduced hardness compared with untreated samples (*p* < 0.05) [49]. In mackerel fillets, HPP did not significantly affect cohesiveness, and mild treatments (100 MPa for 2–5 min) did not significantly alter hardness, chewiness, or springiness. However, more intense treatments (500 MPa for 2–5 min) significantly increased these parameters [60]. In fresh albacore, adhesiveness increased at 200–250 MPa but decreased again at higher pressures (300–500 MPa) [53]. Increased hardness above 200 MPa has been attributed to proteolytic enzyme inactivation combined with pressure-induced protein aggregation, resulting in denser muscle structures [54].

The reduction in hardness observed in the present study may be explained by pressure-induced degradation of structural proteins, particularly myosin and actin. potentially facilitated by proteolytic enzymes released from cells damaged during pressurization [61]. Moderate pressures (<400 MPa) may activate endogenous enzymes such as cathepsins B and L, accelerating protein breakdown [48,49]. Thus, fish tenderization during HPP may result from both direct structural protein disruption and enzymatic activity [49,61]. Although the effects of HPP on TPA parameters vary across studies, 300 MPa appears to represent a critical threshold beyond which texture modifications become more pronounced. In the fish industry, fillet softening is associated with deterioration and a significant quality loss [37]. Therefore, the significant reduction in hardness observed in sea bream fillets after HPP treatment should be considered a negative quality attribute rather than an improvement. Fish are highly susceptible to lipid oxidation due to their high content of polyunsaturated fatty acids, heme-proteins such as hemoglobin, transition metals, and oxidative enzymes [40]. In species with relatively high lipid content, such as cultured sea bream (~13% wet basis), lipid oxidation is a key parameter affecting quality and shelf life. The effect of HPP on lipid oxidation is multifactorial and depends on processing parameters (pressure, time, temperature), intrinsic fish characteristics, and extrinsic factors such as harvesting conditions, pre-processing (e.g., filleting or smoking), and storage conditions [37,40,45].

In agreement with previous studies, treated fillets showed significantly lower TBARS values (*p* < 0.05) compared with controls, and values remained well below the sensory acceptability threshold of 1 mg MDA/kg product. Sea bass treated at pressures above 300 MPa also exhibited significantly lower TBARS values compared with samples processed at 100–200 MPa [54], suggesting that higher pressures may inhibit the formation of secondary lipid oxidation products. However, data from previous studies on the effect of HPP on lipid oxidation in aquatic products remain inconsistent [62].

A preservative effect was reported for Atlantic salmon (*Salmo salar*), where treatment at 300 MPa inhibited lipid oxidation compared with untreated samples and those treated at 150 MPa [63]. The authors suggested that pressure-induced modifications of membrane structure may reduce phospholipid susceptibility to oxidation. Conversely, increased lipid oxidation has been observed in cod (*Gadus morhua*) treated at 400 and 800 MPa for 20 min [41], as well as in carp (*Cyprinus carpio*) and turbot (*Scophthalmus maximus*) treated at 100–200 MPa [41,64,65].

Lipid oxidation is a complex process involving simultaneous formation and degradation of oxidative compounds. Since sea bream is characterized by a low concentration of iron-containing proteins, acting as pro-oxidants, the low TBARS values observed at 200 MPa may be associated with the inactivation of endogenous pro-oxidative enzymes such as lipase and lipoxygenase [54,66].

### 4.3. NIR Discrimination

NIR spectroscopy is an effective tool in numerous industrial sectors (foods, cosmetics, pharmaceuticals, etc.), due to its advantages: speed, simplicity, and reproducibility of analyses. Furthermore, its combination with chemometrics offers the possibility of extracting extensive information from highly complex data sets.

In the present study, the effects of HPP treatment on the fish matrix were investigated through spectroscopic analysis. The spectral profiles of fish fillets subjected to high-pressure processing exhibited clear and detectable modifications. Multivariate analysis, performed using both unsupervised and supervised approaches, revealed treatment-related spectral variations. Specifically, the PCA score plot (Figure 4) showed that the first principal component (PC1) accounted for 86% of the total variance, efficiently separating the T samples from the NT samples. The corresponding loading plot revealed that the separation along the PC1 axis was primarily driven by the 900–930 nm spectral region, associated with the lipid and protein structural features. Furthermore, the loading plot for PC2 showed a significant peak around 1450 nm region, associated with the water absorption band. The separation between T and NT samples became more pronounced with increasing HPP pressure, suggesting progressive physicochemical modifications. Overall, the PCA indicated that both the structural and the aqueous components were the most affected by the HPP treatment, consistent with the physicochemical changes reported in the literature and discussed in the previous section.

Regarding supervised classification, LDA requires a sample size significantly larger than the number of variables to ensure robust statistical performance. To retain the full spectral range while meeting this requirement, the three replicate spectra acquired from each fillet were treated as independent observations for model development. Both LDA and SVM approaches produced similar results and were able to predict correctly T and NT fish with high performance in terms of sensitivity (>96%), specificity (>96%), and accuracy (>98%). Therefore, the chemometric analyses confirmed that HPP-treated and untreated samples could be effectively distinguished. Supervised classification achieved excellent accuracy in discriminating treated from untreated samples. These results demonstrate the potential of NIR spectroscopy combined with multivariate analyses as a rapid, non-destructive tool for verifying HPP treatment and supporting seafood safety management. Although Linear Discriminant Analysis (LDA) and Support Vector Machine (SVM) showed satisfactory classification performance, future studies could investigate the application of additional chemometric and machine learning approaches, such as Partial Least Squares–Discriminant Analysis (PLS-DA) or other advanced algorithms, to further assess the robustness and predictive capability of the proposed NIR-based methodology.

## 5. Conclusions

This study demonstrated that HPP represents a promising non-thermal strategy for the inactivation of *Anisakis* spp. larvae in gilthead sea bream fillets. Under the tested conditions, treatment at 200 MPa for 5 min achieved complete larval inactivation, supporting the potential of HPP as an alternative to conventional freezing for parasite control in fish intended for raw or minimally processed consumption.

However, HPP at 200 MPa induced significant modifications in several physicochemical properties of the fillets, particularly in color and texture, suggesting that the selection of pressure–time conditions should balance parasitological safety with the preservation of product quality.

Furthermore, NIR spectroscopy coupled with multivariate analysis can effectively discriminate HPP-treated from untreated fillets with high accuracy, highlighting its potential as a rapid, non-destructive tool for on-site verification within seafood control systems.

Despite these promising findings, the study was limited to a single fish species and specific processing conditions. Further research is needed to validate these results in other commercially relevant fish species, assess sensory acceptance, and optimize processing parameters to mitigate color and texture changes. In addition, the high capital investment required for HPP systems remains a constraint for widespread industrial adoption, highlighting the need for cost–benefit assessments and scalable implementation strategies.

Overall, the combined application of HPP and portable NIR spectroscopy offers an innovative and integrated approach to improve seafood safety while supporting traceability and quality control in the fish industry.

## Figures and Tables

**Table 1:** Distribution of fish fillets between the two experimental groups.

	Group 1	Group 2	
**Treatment**	**n of Samples for NIR Spectroscopy Analysis**	**n of Samples for NIR Spectroscopy Analysis**	**n Samples for Physicochemical Characteristics**
no treatment	30	90	20 + 10 (TBA)
200 MPa at 5 min	10	60	20 + 10 (TBA)
300 MPa for 1 min	10		
300 MPa for 3 min	10		
600 MPa for 5 min	10		

**Table 2:** Effects of high-pressure processing on the physicochemical characteristics of gilthead sea bream fillets.

Parameters	NT	T	SEM	*p*-Value
pH	6.24	6.31	0.007	**0.001**
Electrical Conductivity, μS/cm	1364.35	1381.10	13.425	0.536
Activity Water	0.941	0.914	0.007	0.056
Purge loss, (%)	3.79	5.69	0.252	**0.001**
Centrifugation loss, (%)	11.33	14.38	0.402	**0.001**
Water Holding Capacity, (%)	82.78	78.10	0.652	**0.001**
Lightness, L*	50.37	71.41	0.290	**0.001**
Redness, a*	−1.95	−2.88	0.058	**0.001**
Yellowness, b*	2.05	6.32	0.187	**0.001**
Chroma, C*	2.92	7.00	0.145	**0.001**
Hue angle, h°	136.21	115.58	1.863	**0.001**
Whiteness Index, WI	50.28	70.55	0.292	**0.001**
Water, (%)	65.81	65.91	0.345	0.889
Protein, (%)	19.62	19.73	0.210	0.785
Ether Extract, (%)	13.36	13.03	0.293	0.572
Ash, (%)	1.21	1.19	0.012	0.408
TBARS, mg MDA/kg fish tissue	2.52	0.56	0.379	**0.019**
Hardness, N	4.60	3.68	0.092	**0.001**
Springiness	0.721	0.90	0.011	**0.001**
Adhesiveness, Nxsec	−0.262	−0.304	0.016	0.190
Cohesiveness	0.319	0.343	0.005	**0.022**
Gumminess; N	1.469	1.26	0.036	**0.005**
Chewiness, N	1.060	1.13	0.032	0.311

Data are presented as Least-Square Means. SEM = standard error of the mean derived from the pooled residual mean square of the ANOVA model. Statistically significant *p* values (*p* < 0.05) are highlighted in bold.

**Table 3:** Classification performances for binary classes: treated/not treated. All values are expressed as percentages.

		Sensitivity %	Specificity %	Accuracy %
LDA	training	99.2	97.5	98.2
	validation	100	96.7	98.3
SVM	training	98.4	98.0	98.2
	validation	96.7	100	98.3

## Data Availability

The datasets presented in this article are not readily available because the data are part of an ongoing study. Requests to access the datasets should be directed the corresponding author.

## References

[1] Mol S., Coşansu S. (2022). Seafood Safety, Potential Hazards and Future Perspective. Turk. J. Fish. Aquat. Sci..

[2] Bruschi F., Gómez-Morales M.A. (2017). Parasites. Foodborne Diseases.

[3] Baird F.J., Gasser R.B., Jabbar A., Lopata A.L. (2014). Foodborne Anisakiasis and Allergy. Mol. Cell. Probes.

[4] Aibinu I.E., Smooker P.M., Lopata A.L. (2019). Anisakis Nematodes in Fish and Shellfish- from Infection to Allergies. Int. J. Parasitol. Parasites Wildl..

[5] Ozuni E., Vodica A., Castrica M., Brecchia G., Curone G., Agradi S., Miraglia D., Menchetti L., Balzaretti C.M., Andoni E. (2021). Prevalence of Anisakis Larvae in Different Fish Species in Southern Albania: Five-Year Monitoring (2016–2020). Appl. Sci..

[6] Caldeira A.J.R., Pereira Alves C.P., Santos M.J. (2021). Anisakis Notification in Fish: An Assessment of the Cases Reported in the European Union Rapid Alert System for Food and Feed (RASFF) Database. Food Control.

[7] Herrador Z., Daschner Á., Perteguer M.J., Benito A. (2019). Epidemiological Scenario of Anisakidosis in Spain Based on Associated Hospitalizations: The Tip of the Iceberg. Clin. Infect. Dis..

[8] Allende A., Alvarez-Ordóñez A., Bortolaia V., Bover-Cid S., De Cesare A., Dohmen W., Guillier L., Herman L., Jacxsens L., Nauta M. (2024). Re-Evaluation of Certain Aspects of the EFSA Scientific Opinion of April 2010 on Risk Assessment of Parasites in Fishery Products, Based on New Scientific Data. Part 2. EFSA J..

[9] Guardone L., Armani A., Nucera D., Costanzo F., Mattiucci S., Bruschi F. (2018). Human Anisakiasis in Italy: A Retrospective Epidemiological Study over Two Decades. Parasite.

[10] Brutti A., Rovere P., Cavallero S., D’Amelio S., Danesi P., Arcangeli G. (2010). Inactivation of Anisakis Simplex Larvae in Raw Fish Using High Hydrostatic Pressure Treatments. Food Control.

[11] Koutsoumanis K., Allende A., Alvarez-Ordóñez A., Bover-Cid S., Chemaly M., De Cesare A., Herman L., Hilbert F., Lindqvist R., Nauta M. (2024). Re-Evaluation of Certain Aspects of the EFSA Scientific Opinion of April 2010 on Risk Assessment of Parasites in Fishery Products, Based on New Scientific Data. Part 1: ToRs1–3. EFSA J..

[12] Tavares J., Martins A., Fidalgo L.G., Lima V., Amaral R.A., Pinto C.A., Silva A.M., Saraiva J.A. (2021). Fresh Fish Degradation and Advances in Preservation Using Physical Emerging Technologies. Foods.

[13] Zhao Y.M., de Alba M., Sun D.W., Tiwari B. (2019). Principles and Recent Applications of Novel Non-Thermal Processing Technologies for the Fish Industry—A Review. Crit. Rev. Food Sci. Nutr..

[14] Roobab U., Fidalgo L.G., Arshad R.N., Khan A.W., Zeng X.A., Bhat Z.F., Bekhit A.E.D.A., Batool Z., Aadil R.M. (2022). High-Pressure Processing of Fish and Shellfish Products: Safety, Quality, and Research Prospects. Compr. Rev. Food Sci. Food Saf..

[15] Koutsoumanis K., Alvarez-Ordóñez A., Bolton D., Bover-Cid S., Chemaly M., Davies R., De Cesare A., Herman L., Hilbert F., Lindqvist R. (2022). The Efficacy and Safety of High-Pressure Processing of Food. EFSA J..

[16] Matser A.M., Stegeman D., Kals J., Bartels P.V. (2000). Effects of High Pressure on Colour and Texture of Fish. High Press. Res..

[17] Ali A., Wei S., Ali A., Khan I., Sun Q., Xia Q., Wang Z., Han Z., Liu Y., Liu S. (2022). Research Progress on Nutritional Value, Preservation and Processing of Fish—A Review. Foods.

[18] Beć K.B., Grabska J., Huck C.W. (2022). Miniaturized NIR Spectroscopy in Food Analysis and Quality Control: Promises, Challenges, and Perspectives. Foods.

[19] Nieto-Ortega S., Lara R., Foti G., Melado-Herreros Á., Olabarrieta I. (2023). Applications of Near-Infrared Spectroscopy (NIRS) in Fish Value Chain. Infrared Spectroscopy—Perspectives and Applications.

[20] Moncayo S., Manzoor S., Navarro-Villoslada F., Caceres J.O. (2015). Evaluation of Supervised Chemometric Methods for Sample Classification by Laser Induced Breakdown Spectroscopy. Chemom. Intell. Lab. Syst..

[21] Lv H., Xu W., You J., Xiong S. (2017). Classification of Freshwater Fish Species by Linear Discriminant Analysis Based on near Infrared Reflectance Spectroscopy. J. Near Infrared Spectrosc..

[22] Lee K.H., Park S.Y., Ha S. (2016). Do Inactivation of Anisakis Simplex L3 in the Flesh of White Spotted Conger (*Conger myriaster*) by High Hydrostatic Pressure and Its Effect on Quality. Food Addit. Contam. Part A Chem. Anal. Control Expo. Risk Assess.

[23] Ortiz A., León L., Ramírez M.R., Tejerina D. (2025). Near-Infrared Spectroscopy as a Tool for the Traceability Control of High-Quality Iberian Dry-Cured Meat Products. Foods.

[24] Commission Internationale de l’Éclairage (CIE) (1976). Recommendations on Uniform Colour Spaces-Colour Difference Equations, Psychometric Colour Terms (Supplement No. 2 to CIE Publication No. 15).

[25] Ramirez-Suarez J.C., Morrissey M.T. (2006). Effect of High Pressure Processing (HPP) on Shelf Life of Albacore Tuna (*Thunnus alalunga*) Minced Muscle. Innov. Food Sci. Emerg. Technol..

[26] Malcolm C. (1982). Bourne food science and technology: A series of monographs. Food Texture and Viscosity.

[27] Eide O., Børresen T., Strøm T. (1982). Minced Fish Production from Capelin (*Mallotus villosus*). A New Method for Gutting, Skinning and Removal of Fat from Small Fatty Fish Species. J. Food Sci..

[28] Grau R., Hamm R. (1953). Eine Einfache Methode Zur Bestimmung Der Wasserbindung Im Muskel. Naturwissenschaften.

[29] Szmańko T., Lesiów T., Górecka J. (2021). The Water-Holding Capacity of Meat: A Reference Analytical Method. Food Chem..

[30] Wiroonsri P., Wattanachant S. (2025). Electrical Conductivity as a Precise Method for Salt Content Estimation in Raw and Cooked Tuna Meat. J. Food Compos. Anal..

[31] AOAC International (2000). Official Methods of Analysis of AOAC International.

[32] Gai F., Gasco L., Ortoffi M., Gonzáles-Rodríguez Á., Parisi G. (2014). Effects of Green Tea Natural Extract on Quality Parameters and Lipid Oxidation during Storage of Tench (*Tinca tinca*) Fillets. J. Appl. Ichthyol..

[33] Ballabio D., Consonni V. (2013). Classification Tools in Chemistry. Part 1: Linear Models. PLS-DA. Anal. Methods.

[34] Tejada M., Huidobro A. (2002). Quality of Farmed Gilthead Seabream [*Sparus aurata*] during Ice Storage Related to the Slaughter Method and Gutting. Eur. Food Res. Technol..

[35] Erkan N., Üretener G. (2010). The Effect of High Hydrostatic Pressure on the Microbiological, Chemical and Sensory Quality of Fresh Gilthead Sea Bream (*Sparus aurata*). Eur. Food Res. Technol..

[36] Giannoglou M., Dimitrakellis P., Efthimiadou A., Gogolides Ε., Katsaros G. (2021). Comparative Study on the Effect of Cold Atmospheric Plasma, Ozonation, Pulsed Electromagnetic Fields and High-Pressure Technologies on Sea Bream Fillet Quality Indices and Shelf Life. Food Eng. Rev..

[37] Truong B.Q., Buckow R., Stathopoulos C.E., Nguyen M.H. (2015). Advances in High-Pressure Processing of Fish Muscles. Food Eng. Rev..

[38] Molina-Garci’a A.D., Garci’a G., Sanz P.D. (2002). Anisakis Simplex Larva Killed by High-Hydrostatic-Pressure Processing. J. Food Prot..

[39] Aganovic K., Hertel C., Vogel R.F., Johne R., Schlüter O., Schwarzenbolz U., Jäger H., Holzhauser T., Bergmair J., Roth A. (2021). Aspects of High Hydrostatic Pressure Food Processing: Perspectives on Technology and Food Safety. Compr. Rev. Food Sci. Food Saf..

[40] de Oliveira F.A., Neto O.C., dos Santos L.M.R., Ferreira E.H.R., Rosenthal A. (2017). Effect of High Pressure on Fish Meat Quality—A Review. Trends Food Sci. Technol..

[41] Angsupanich K., Ledward D.A. (1998). High Pressure Treatment Effects on Cod (*Gadus morhua*) Muscle. Food Chem..

[42] Truong B.Q., Buckow R., Nguyen M.H., Stathopoulos C.E. (2016). High Pressure Processing of Barramundi (*Lates calcarifer*) Muscle before Freezing: The Effects on Selected Physicochemical Properties during Frozen Storage. J. Food Eng..

[43] Huff-Lonergan E., Lonergan S.M. (2005). Mechanisms of Water-Holding Capacity of Meat: The Role of Postmortem Biochemical and Structural Changes. Meat Sci..

[44] Lakshmanan V., Fritz A., Smith T., Hondl K., Stumpf G. (2007). An Automated Technique to Quality Control Radar Reflectivity Data. J. Appl. Meteorol. Climatol..

[45] Campus M., Addis M.F., Cappuccinelli R., Porcu M.C., Pretti L., Tedde V., Secchi N., Stara G., Roggio T. (2010). Stress Relaxation Behaviour and Structural Changes of Muscle Tissues from Gilthead Sea Bream (*Sparus aurata* L.) Following High Pressure Treatment. J. Food Eng..

[46] Cropotova J., Mozuraityte R., Standal I.B., Ojha S., Rustad T., Tiwari B. (2020). Influence of High-Pressure Processing on Quality Attributes of Haddock and Mackerel Minces during Frozen Storage, and Fishcakes Prepared Thereof. Innov. Food Sci. Emerg. Technol..

[47] Hedges N.D., Goodband R.M. The Influence of High Hydrostatic Pressure on the Water-Holding Capacity of Fish Muscle. Proceedings of the First Joint Trans-Atlantic Fisheries Technology Conference (TAFT), 33rd WEFTA and 48th AFTC Meetings.

[48] Teixeira B., Marques A., Mendes R., Gonçalves A., Fidalgo L., Oliveira M., Saraiva J.A., Nunes M.L. (2014). Effects of High-Pressure Processing on the Quality of Sea Bass (*Dicentrarchus labrax*) Fillets During Refrigerated Storage. Food Bioproc. Tech..

[49] Chéret R., Chapleau N., Delbarre-Ladrat C., Verrez-Bagnis V., de Lamballerie M. (2005). Effects of High Pressure on Texture and Microstructure of Sea Bass (*Dicentrarchus labrax* L.) Fillets. J. Food Sci..

[50] Bolumar T., Orlien V., Sikes A., Aganovic K., Bak K.H., Guyon C., Stübler A., de Lamballerie M., Hertel C., Brüggemann D.A. (2021). High-pressure Processing of Meat: Molecular Impacts and Industrial Applications. Compr. Rev. Food Sci. Food Saf..

[51] Yagiz Y., Kristinsson H.G., Balaban M.O., Marshall M.R. (2007). Effect of High Pressure Treatment on the Quality of Rainbow Trout (*Oncorhynchus mykiss*) and Mahi Mahi (*Coryphaena hippurus*). J. Food Sci..

[52] Tsironi T., Anjos L., Pinto P.I.S., Dimopoulos G., Santos S., Santa C., Manadas B., Canario A., Taoukis P., Power D. (2019). High Pressure Processing of European Sea Bass (*Dicentrarchus labrax*) Fillets and Tools for Flesh Quality and Shelf Life Monitoring. J. Food Eng..

[53] Cartagena L., Puértolas E., Martínez de Marañón I. (2020). Evolution of Quality Parameters of High Pressure Processing (HPP) Pretreated Albacore (*Thunnus alalunga*) during Long-Term Frozen Storage. Innov. Food Sci. Emerg. Technol..

[54] Tsevdou M., Gogou E., Taoukis P. (2019). High Hydrostatic Pressure Processing of Foods. Green Food Processing Techniques.

[55] Ayala M.D., Santaella M., Martínez C., Periago M.J., Blanco A., Vázquez J.M., Albors O.L. (2011). Muscle Tissue Structure and Flesh Texture in Gilthead Sea Bream, *Sparus aurata* L., Fillets Preserved by Refrigeration and by Vacuum Packaging. LWT Food Sci. Technol..

[56] Lullien-Pellerin V., Balny C. (2002). High-Pressure as a Tool to Study Some Proteins’ Properties: Conformational Modification, Activity and Oligomeric Dissociation. Innov. Food Sci. Emerg. Technol..

[57] Messens W., Van Camp J., Huyghebaert A. (1997). The Use of High Pressure to Modify the Functionality of Food Proteins. Trends Food Sci. Technol..

[58] Gokul Nath K., Pandiselvam R., Sunil C.K. (2023). High-Pressure Processing: Effect on Textural Properties of Food- A Review. J. Food Eng..

[59] Puértolas E., Lavilla M. (2020). HPP in Seafood Products: Impact on Quality and Applications. Present and Future of High Pressure Processing.

[60] de Alba M., Pérez-Andrés J.M., Harrison S.M., Brunton N.P., Burgess C.M., Tiwari B.K. (2019). High Pressure Processing on Microbial Inactivation, Quality Parameters and Nutritional Quality Indices of Mackerel Fillets. Innov. Food Sci. Emerg. Technol..

[61] Christensen L.B., Hovda M.B., Rode T.M. (2017). Quality Changes in High Pressure Processed Cod, Salmon and Mackerel during Storage. Food Control.

[62] Aubourg S.P. (2018). Impact of High-pressure Processing on Chemical Constituents and Nutritional Properties in Aquatic Foods: A Review. Int. J. Food Sci. Technol..

[63] Yagiz Y., Kristinsson H.G., Balaban M.O., Welt B.A., Ralat M., Marshall M.R. (2009). Effect of High Pressure Processing and Cooking Treatment on the Quality of Atlantic Salmon. Food Chem..

[64] Sequeira-Munoz A., Chevalier D., LeBail A., Ramaswamy H.S., Simpson B.K. (2006). Physicochemical Changes Induced in Carp (*Cyprinus carpio*) Fillets by High Pressure Processing at Low Temperature. Innov. Food Sci. Emerg. Technol..

[65] Chevalier D., Le Bail A., Ghoul M. (2001). Effects of High Pressure Treatment (100–200 MPa) at Low Temperature on Turbot (*Scophthalmus maximus*) Muscle. Food Res. Int..

[66] Pazos M., Méndez L., Fidalgo L., Vázquez M., Antonio Torres J., Aubourg S.P., Saraiva J.A. (2015). Effect of High-Pressure Processing of Atlantic Mackerel (*Scomber scombrus*) on Biochemical Changes During Commercial Frozen Storage. Food Bioproc. Tech..

